# Effect of a sodium hypochlorite mouthwash on plaque and clinical parameters of periodontal disease‐a systematic review

**DOI:** 10.1111/idh.12510

**Published:** 2021-07-19

**Authors:** Ahsan Mehran Hussain, G. A. (Fridus) van der Weijden, Dagmar Else Slot

**Affiliations:** ^1^ Department of Periodontology Academic Center for Dentistry Amsterdam (ACTA) University of Amsterdam and Vrije Universiteit Amsterdam The Netherlands

**Keywords:** dental plaque, mouthwashes, periodontal health, sodium hypochlorite, systematic review

## Abstract

**Objective:**

The present study aimed to establish the efficacy of sodium hypochlorite mouthwash (NaOCl‐MW) compared with a control mouthwash on plaque and clinical parameters of periodontal disease.

**Methods:**

MEDLINE‐PubMed, Embase and Cochrane‐CENTRAL databases were searched for clinical trials on patients with gingivitis or periodontitis that assessed the effect of NaOCl‐MW in comparison with a negative or positive control on plaque index (PI), gingival index (GI), and bleeding index (BI) scores and probing pocket depth (PPD). Data were extracted from the eligible studies.

**Results:**

Seven eligible papers were retrieved, which together represented six clinical trials. The studies showed considerable heterogeneity regarding methodological and clinical aspects that did not permit a meta‐analysis. Two of the three studies in which NaOCl‐MW was compared with a negative control showed that NaOCl‐MW significantly reduced PI, GI and BI, and no effect was found on PPD. In three studies, NaOCl‐MW was assessed using chlorhexidine mouthwash (CHX‐MW) as a positive control; no difference was found for GI and BI. One of the three comparisons showed a statistically significant PI score favouring NaOCl‐MW. One study measured PPD and found it to be significant in favour of NaOCl‐MW.

**Conclusions:**

Studies with a negative control group provided very weak quality evidence for a very small beneficial effect of NaOCl‐MW on PI, GI and BI scores. Studies with a positive control group provided very weak quality evidence that NaOCl‐MW had a similar effect as CHX‐MW on PI, GI and BI scores. The outcome for PPD was inconclusive.

## 1 INTRODUCTION

Gingivitis and periodontitis are the most common soft tissue oral diseases, and dental caries is the most predominant hard tissue oral disease in humans worldwide.^1^, ^2^ Gingivitis and dental caries are both primarily induced by an undisturbed accumulation of dental plaque that adheres to the intraoral hard surfaces. Dental plaque consists of a broad range of bacteria, their products, epithelial shedding's and food debris. When gingivitis is left untreated, it may progress to periodontitis. Attachment loss due to periodontitis can eventually lead to tooth loss, which has an adverse effect on chewing, speech, quality of life, and self‐confidence and may have systemic inflammatory consequences.^3^, ^4^

To maintain a healthy periodontium or treat periodontal disease, dental plaque needs to be daily and meticulously removed. Mechanical plaque removal with a manual or electric toothbrush is the first choice of oral hygiene device to reduce dental plaque.^3^ Interproximal cleaning devices are also recommended as adjunct to toothbrushes.^5^ However, there is substantial evidence that efficient mechanical plaque control is not achieved by most individuals of the general population.^6^, ^7^ Several reasons are proposed, including limited time of usage and limited use of interdental cleaning devices. Therefore, chemical plaque control could be considered as a part of daily home care measures.^3^, ^8^ Adjunctive anti‐microbial agents are available to consumers in the form of mouthwash and toothpaste/gel.

Chlorhexidine mouthwash (CHX‐MW) is a regularly advised chemical plaque control product and is considered as a gold standard. It has both bactericidal and bacteriostatic properties. There is a large body of evidence that supports the effectiveness of CHX‐MW, showing that it can significantly improve parameters of plaque and gingivitis.^9^, ^10^ However, CHX also has some side effects such as stimulation of calculus formation, hypogeusia, burning sensation, hypersensitivity and extrinsic tooth staining from long‐term use.^9^ These side effects may have a negative effect on patient compliance in using this mouthwash. Therefore, dental care professionals commonly do not advise the use of CHX‐MW for an extended period.^9^, ^11^, ^12^

Sodium hypochlorite (NaOCl) has been used for various purposes around the world as a strong anti‐microbial agent. It is used in hospitals, animal facilities and potable water supplies, and it serves as a food additive and bleaching agent.^13^ In dentistry, it is employed, in concentrations of 1%–6%, as the favoured root canal irrigant for treating endodontic infections.^14^, ^15^ In water, NaOCl settles an equilibrium with Na^+^, OH^−^ and hypochlorous acid (HOCl). HOCl is a weak acid that further dissociates into H^+^ and hypochlorite ion (OCl^−^). HOCl has stronger anti‐microbial abilities than OCl^−^. This can partly be explained by the fact that pathogenic microorganisms by nature have negatively charged cell walls. These cell walls can only be penetrated by neutrally charged HOCl and not by OCl^−^.^16^, ^17^ Hypochlorous acid is capable of penetrating the polysaccharide plaque matrix and oxidizing and disrupting the cell wall, cell membrane and various macromolecules of microorganisms, such as proteins, nucleotides and lipids.^18^ NaOCl is naturally produced in activated inflammatory cells such as neutrophils and macrophages and plays a crucial anti‐microbial role in the innate immune system.^19^ Thus, it does not evoke allergic reactions; is not a carcinogen, mutagen, teratogen, or cytotox; and has a century‐long safety record.^20^ Histologically, no damage was observed to periodontal connective tissues after applying 6% NaOCl subgingivally.^21^ It also does not increase the risk of resistance development because it attacks multiple components of infectious agents. In 1984, The American Dental Association Council on Dental Therapeutics designated 0.1% NaOCl as a mild antiseptic mouthrinse, and its suggested use is direct application on the mucous membrane.^22^

NaOCl can be used as a mouthwash as it has excellent anti‐microbial properties and is a safe and low‐cost antiseptic agent. Sodium hypochlorite is available in most homes as a household bleach. It has been suggested that patients could dilute inexpensive basic household bleach to reach the recommended concentration.^23^, ^24^ Several studies have shown that it has anti‐microbial activity against the dental plaque microflora and can reduce gingivitis.^25^, ^26^, ^27^ However, there are other scientific studies that do not support this proposition.^28^ Therefore, at present, the results published regarding the effectiveness of NaOCl remain inconclusive.

The purpose of this systematic review was to gather and synthesize all the available scientific literature to investigate and compare the efficacy of NaOCl mouthwash (NaOCl‐MW) with that of control mouthwashes on plaque scores and clinical parameters related to periodontal disease.

## 2 MATERIAL AND METHODS

The preparation and presentation of this systematic review are in accordance with the Cochrane Handbook for Systematic Reviews of Interventions ^29^ and the guidelines of Transparent Reporting of Systematic Reviews and Meta‐Analyses (PRISMA).^30^ A protocol was developed a priori following an initial discussion among the research team members.^31^ This systematic review was registered beforehand at ACTA ETC (protocol number 202093) and PROSPERO (protocol number 236831).

### 2.1 Focus question (PICO)

In patients with gingivitis or periodontitis, what is the effect of rinsing with NaOCl‐MW compared with a control mouthwash on plaque scores and clinical parameters related to periodontal disease?

### 2.2 Search strategy

To retrieve studies concerning the effect of NaOCl‐MW, a structured and comprehensive search strategy was designed. The National Library of Medicine, Washington D.C. (MEDLINE‐PubMed), the Cochrane Central Register of Controlled Trials (CENTRAL) and EMBASE (Excerpta Medical Database by Elsevier) were searched from inception until October 2020. The reference lists of the included studies were manually searched to identify additional potentially relevant studies. Table 1 provides details regarding the search terms used. There were no restrictions on publication date.

**TABLE 1:** Search terms used for the search strategy

The following strategy was used in the search: {<active ingredient>AND <vehicle >}
Search terms used for PubMed‐MEDLINE: {< (MeSH terms) Sodium hypochlorite OR (text words) sodium hypochlorite OR household bleach OR bleach OR NaOCl> AND < (MeSH terms) Mouthwashes OR (text words) mouthwash OR mouthwash* OR mouthrins* OR mouthrinse>}

Note

The asterisk (*) was used as a truncation symbol. The search strategy was customized according each of the three database searched.

### 2.3 Screening and selection

For all studies obtained from the search, the title and abstract (when available) were judged independently by two reviewers (AMS and DES) using the Rayyan^32^ web application. Studies that potentially fulfilled the inclusion criteria for full‐text reading or for which the title and abstract provided inadequate information to make a clear assessment were selected. After reading the full texts, the studies were categorized as ‘definitely eligible’, ‘definitely not eligible’ or ‘questionable’. Disagreements concerning eligibility were resolved by consensus or—if disagreement persisted—by arbitration by a third reviewer (GAW). The papers that fulfilled all of the inclusion criteria were processed for data extraction. Attempts were made to contact the authors of the included publications to request additional data or information if the paper was unclear.

The inclusion criteria were as follows:

1. Randomized controlled trials (RCTs) or controlled clinical trials (CCTs)
2. Published in the English Language
3. Trials conducted with human participants in good general health (no systemic disorders)
4. Patients with gingivitis or periodontitis
5. Intervention: NaOCl‐MW
6. Comparison with negative control: placebo rinse or water
7. Comparison with positive control: CHX or essential oil (EO) mouthwashes as these are considered effective for plaque control and managing gingival inflammation.^33^
8. Outcome parameters: plaque index (PI), gingival index (GI), and bleeding index (BI) scores and probing pocket depth (PPD)

### 2.4 Assessment of heterogeneity

The following factors were considered to determine the heterogeneity of the outcomes of the different studies: study design, evaluation period, subject characteristics, control groups, NaOCl concentration, mouthwash brand and rinsing procedure.

### 2.5 Methodological quality assessment

The potential risk of bias of the studies included in this review was estimated independently by two reviewers (AMS and DES) using the checklist for RCTs presented in Appendix S1 as proposed by Van der Weijden et al. (2009).^34^ If there was a disagreement between two reviewers, a consensus was achieved through discussion. If there was no consensus after the discussion, the opinion of a third reviewer (GAW) was decisive. In brief, when positive scores were assigned to defined inclusion/exclusion criteria, random allocation, balanced experimental groups, blinding of the patient to the product, blinding of the examiner, identical treatment between groups (except for intervention) and reporting of follow‐up, the study was classified as having a low risk of bias. When the study fulfilled only six of these seven criteria, it was considered to have a moderate risk of bias. If more than one of these seven criteria remained unfulfilled, the article was considered to have a high risk of bias.

### 2.6 Data extraction and analysis

For all the included studies, data extraction was performed by two independent reviewers (AMS and DES) using a custom‐designed data extraction form. Data recorded were based directly on the focus of the research question and also included details of the study population, intervention, comparison, outcome and study characteristics. Means and standard deviations were extracted if available. A consensus was achieved through discussion if there was a disagreement between two reviewers. Any persisting disagreements were resolved by discussion with a third reviewer (GAW). To obtain a summary of the data, a descriptive data presentation was used for all the studies.^35^ It was decided in advance to categorize the NaOCl‐MW studies into either negative control group studies or positive (CHX or EO‐MW) control group studies.^33^ The PI, GI, and BI scores and PPD measurements were taken into account.

### 2.7 Grading the body of evidence

The Grading of Recommendation, Assessment, Development and Evaluation (GRADE) system was used to rank the evidence emerging from this review.^36^, ^37^, ^38^ Two reviewers (AMH and DES) rated the quality of the evidence and the strength and direction of the recommendation according to the strength of the following aspects: risk of bias, consistency of results, directness of evidence, precision, reporting bias and magnitude of effect. Any disagreement between the two reviewers (AMH and DES) was resolved by additional discussion with GAW.

## 3 RESULTS

### 3.1 Search and selection results

**FIGURE 1:**
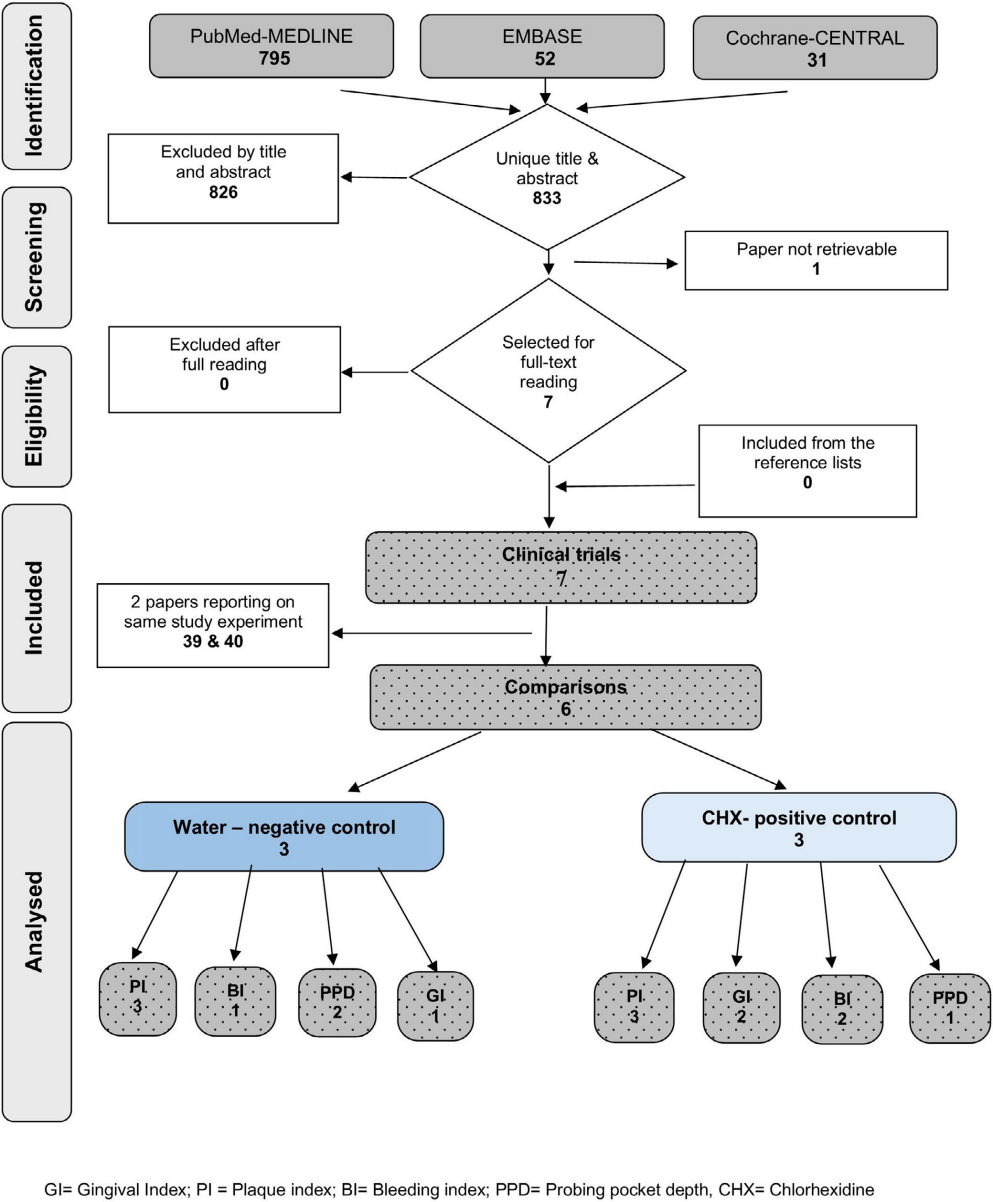
Flow chart of search and selection process and outcome

A search of the MEDLINE‐PubMed, Cochrane‐CENTRAL and EMBASE databases yielded 833 unique papers (Figure 1). Screening of the titles and abstracts resulted in the selection of seven papers for which the full texts were obtained and read in detail. All seven papers were found to be eligible. Manually searching the reference lists of these papers did not yield additional publications. The seven selected papers represented six clinical trials and six comparisons because the papers by Galvan et al. (2014) ^39^ and Gonzales et al. (2015) ^40^ involved the same experiment. Galvan et al. (2014) ^39^ aimed to evaluate the effect of NaOCl‐MW on plaque and gingivitis in patients with periodontitis, and Gonzales et al. (2015) ^40^ published a sub‐analysis of the effect of NaOCl‐MW on bleeding on probing scores in relation to pocket depth measurements. The efficacy of NaOCl‐MW was evaluated in three comparisons (I,^26^ II^28^ and V^39^, ^40^) with a negative control and three comparisons (III,^27^ IV^25^ and VI^41^) with a positive control.

### 3.2 Assessment of heterogeneity

The six clinical trials showed heterogeneity with respect to study design, evaluation period, participants, control groups, mouthwash concentration and brand, rinsing procedure, and assessment parameters. Information regarding the study characteristics is presented in Table 2.

**TABLE 2:** Overview of the included studies characteristics and details including the conclusions of the original publications

Non‐brushing Brushing Gingivitis/ Periodontitis Risk of bias	Authors (year)	Study design, blinding, duration	Participants baseline (end), gender, age (mean/range)	Groups % Instruction	Brands	Conclusions of the original authors
Non‐brushing protocol Experimental gingivitis model High	I De Nardo et al. (2012) ^26^	RCT Parallel Single (investigator)‐blinded 21 days (pre‐experimental 30 days)	44 (40) ♀: 40 ♂:? Mean age: 27.8 (5.6) Age range:?	NaOCl 0.05% (n=20) Water (n=20) 15 ml for 60 s 2x p/day	10% NaOCl from chemical drugstore 5 ml mixed with 995 ml water to obtain 0.05% Fresh solution every 24 h	An oral rinse with 0.05% NaOCl resulted in significant reductions in supragingival biofilm accumulation and gingival inflammation. Diluted NaOCl may represent an efficacious, safe and affordable anti‐microbial agent in the prevention and treatment of periodontal disease.
Brushing protocol Gingivitis Low	II Espindola et al. (2017) ^28^	RCT Parallel Double‐blinded 6 months	32 (28) ♀: 8 ♂: 20 Mean age: 24.7 (5.3) T group 25.1 (6.8) C group Age range:?	NaOCl 0.1% (n=13) Water (n=15) 15 ml for 30 s 2x p/day for 4 weeks Brushing with a manual toothbrush and dental floss for interdental cleaning	2.5% NaOCl (Asfer Industria Quima Ltda., SP, Brazil) Mixed with sterile water to obtain 0.1% Fresh prepared every week	0.1% NaOCl‐MW did not provide additional benefits to full‐mouth ultrasonic debridement in reducing supragingival plaque, gingivitis and/or microbial pathogens.
Brushing protocol Gingivitis Moderate	III Shanker et al. (2018) ^27^	RCT Parallel Single (investigator)‐ blinded 2 weeks	100 (80) ♀:? ♂:? Mean age: 32.85 (16.28) T group 30.88 (10.82) C group Age range:?	NaOCl 0.25% (n=40) CHX 0.2% (n=40) 15 ml for 30 s 2x p/day 30 min after toothbrushing with a manual toothbrush No eating and drinking for ½ h after rinsing	5.25% Clorex® (The Clorex company, USA) 5 ml mixed with 120 ml distilled water to obtain 0.25% Hexidine® (ICPA Health products Ltd., Mumbai, India)	0.25% NaOCl as MW was more efficacious than CHX in the treatment of chronic gingivitis patients.
Brushing protocol Gingivitis High	IV Mishra et al. (2019) ^25^	CCT Parallel Blinding? 21 days	60 (60) ♀:? ♂:? Mean age:? Age range:?	NaOCl 0.5% (n=30) CHX 0.2% (n=30) 10 ml for 60 s 2x p/day No eating and drinking for ½ h after rinsing Brushing with a manual toothbrush	?	0.2% NaOCl‐MW is as effective as 0.2% CHX for the treatment of gingivitis as it is an adjunct to mechanical plaque removal in terms of safety, less side effects, less staining and can be used as a routine mouthwash.
Brushing protocol Periodontitis Moderate	V Galvan et al. (2014) ^39^ & Gonzales et al. (2015) ^40^	RCT Parallel Single (investigator)‐blinded 3 months	30 (12) ♀: 17 ♂: 13 Mean age: 41 Age range:?	NaOCl 0.25% (n=7) Water (n=5) 15 ml for 30 s 2x p/week (wo and sun) No rinsing with water afterwards for at least 10 min Brushing with a manual toothbrush and dental floss for interdental cleaning	6% Clorex® (The Clorex company, USA) 5 ml mixed with 120 ml water to obtain 0.25% Fresh solution at each time of rinsing	A twice‐weekly oral rinse with 0.25% NaOCl produced marked decreases in dental plaque level and bleeding on probing and may constitute a promising new approach to the management of periodontal disease. Twice weekly oral rinsing with diluted bleach (0.25% NaOCl) produced a significant reduction in bleeding on probing, even in deep unscaled pockets. NaOCl constitutes a valuable antiseptic in periodontal self‐care.
Brushing protocol Periodontitis High	VI Singh et al. (2020) ^41^	RCT Parallel Blinding? 6 months	60 ♀:? ♂:? Mean age:? Age range:?	NaOCl 0.05% (n=30) CHX 0.12% (n=30) 2x p/day for 4 weeks Oral hygiene instructions?	2.5% Household bleach 5 ml mixed with 250 ml water to obtain 0.05% ?	NaOCl when prescribed as a twice daily mouthwash can be recommended as a part of the home care regime in patients with chronic periodontitis. It is more cost‐effective, easily available and can be beneficial to the troops in difficult terrains and extremes of climates, where oral healthcare facilities are not easily accessible.

Abbreviations: CCT, controlled clinical trial; C‐group, control group; CHX, chlorhexidine; NaOCl, sodium hypochlorite; RCT, randomized controlled tial; T‐group, test group.

### 3.3 Study design and participant characteristics

Five of the selected comparisons were parallel‐design RCTs (I,^26^ II,^28^ III,^27^ V^39^, ^40^ and VI^41^), and one comparison was a parallel‐design CCT (IV^25^). Shanker et al. (III^27^) mention that their study had a case‐control design, but they had randomly distributed the patients into test and control groups; consequently, the trial was assumed to be an RCT. Five comparisons (II,^28^ III,^27^ IV,^25^ V^39^, ^40^ and VI^41^) used mouthwash as an adjunct to self‐performed daily oral hygiene, with the study duration ranging from 2 weeks ^27^ to 6 months.^28^, ^41^ In three comparisons (III,^27^ IV^25^ and V^39^, ^40^), participants used a mouthrinse for the entire course of the study. In the study by Espindola et al. (II^28^) and Singh et al. (VI^41^), the patients used a mouthrinse for 4 weeks, and the study duration was 6 months. One study (I^26^) used an experimental gingivitis model ^42^ with mouthrinse use for 21 days, during which the subjects were instructed to abstain from all other oral hygiene measures. In four of the six comparisons (II,^28^ III,^27^ IV^25^ and V^39^, ^40^), the participants were recruited at the department of periodontology at a school of dentistry, and in one (I^26^), the patients were recruited at a men's prison. For the study by Singh et al. (VI^41^), patients were recruited from two different dental centres. All participants included in the final selection of studies were healthy adults. Singh et al. (VI^41^) did not mention any eligibility criteria. Three studies (II,^28^ III^27^ and IV^25^) included only patients with gingivitis—the inclusion criteria used were >10% of sites with bleeding on probing, no probing depth and clinical attachment loss <3 mm (II^28^), chronic marginal gingivitis (III^27^), bleeding index >50% and mild‐to‐moderate gingivitis (IV^25^). Two comparisons (V^39^, ^40^ and VI^41^) specifically included patients with periodontitis, where the participants had at least four teeth with a PPD of ≥6 mm (V^39^, ^40^). One comparison (I^26^) included patients with healthy gingivae or slight periodontitis with clinical attachment loss of ≤2 mm.

In two comparisons (I^26^ and III^27^), a complete oral prophylaxis was performed to bring the gingival status to healthy levels at baseline in the pre‐experimental period, and in two comparisons (II^28^ and VI^41^), full‐mouth supra‐ and subgingival ultrasonic treatment was provided before the participants started using the mouthwash. In comparison V,^39^, ^40^ participants received subgingival irrigation with either 0.25% NaOCl or water at baseline and after 2 weeks in addition to self‐performed mouth rinsing.

### 3.4 NaOCl concentrations

The NaOCl concentration in the mouthwashes used differed across the studies. A concentration of 0.25% was used in comparisons III^27^ and V,^39^, ^40^ a concentration of 0.05% in studies I^26^ and VI,^41^ a concentration of 0.1% in study II^28^ and a concentration of 0.5% in study IV.^25^ In studies III,^27^ IV^25^ and VI,^41^ the concentration of CHX was 0.2%. Studies I,^26^ IV^25^ and VI^41^ did not mention the brand of the CHX‐MW product.

### 3.5 Rinsing regimen

The rinsing procedure was set at twice daily for 60 s in studies I^26^ and IV,^25^ twice daily for 30 s in studies II^28^ and III,^27^ and twice per week for 30 s in comparison V.^39^, ^40^ In study VI,^41^ participants used the mouthrinse twice per week, but the rinsing duration was not specified. The studies used 15 mL of rinsing solution, except study IV,^25^ which used 10 mL, and study VI,^41^ which did not report the rinsing volume. In all brushing studies (II,^28^ III,^27^ IV^25^ and V^39^, ^40^), patients were instructed to brush twice daily with a manual toothbrush, and in studies II^28^ and V,^39^, ^40^ the patients also used dental floss for interproximal cleaning. Study VI^41^ did not provide details about oral hygiene instructions. Participants in studies III^27^ and IV^25^ were asked not to eat or drink for 30 min after mouth rinsing, and those in study V^39^, ^40^ were asked not to rinse with water for at least 10 min.

### 3.6 Indices and modifications

A variety of indices and their modifications were used to score the outcome parameters. For plaque, two studies (I^26^ and II^28^) used the Quigley & Hein plaque index ^43^ as modified by Turesky et al.,^44^ two studies (IV^25^ and VI^41^) used the Silness & Löe ^45^ plaque index, and three studies only scored plaque as present or absent at six sites (II^28^) or two sites (V^39^, ^40^). For gingival inflammation, the modified Löe & Silness gingival index ^45^ was used in two studies (I^26^ and IV^25^), and the modified gingival index by Lobene et al. ^46^ was used in one study (III^27^). Bleeding on probing tendency was scored using the gingival bleeding index by Ainamo and Bay ^47^ in comparison IV^25^ and gingival sulcus bleeding index by Mühlemann & Son ^48^ in comparison VI^41^ and by probing to the bottom of the pocket at four and six sites in comparison I^26^ and comparisons II^28^ and V,^39^, ^40^ respectively. In comparisons II,^28^ V^39^, ^40^ and VI,^41^ the PPD was measured using the probe at six sites around the teeth.

### 3.7 Methodological quality assessment

The potential risk of bias was estimated on the basis of methodological quality aspects of the included papers using the checklist presented in Appendix S1. Based on the summary of the proposed criteria, the estimated risk of bias was low for comparison II,^28^ moderate for comparisons III^27^ and V,^39^, ^40^ and high for comparisons I,^26^ IV^25^ and VI.^41^

### 3.8 Study outcomes

The results reported by the included studies for PI, GI, BI and PPD are presented in Appendix S2. A meta‐analysis could not be performed owing to missing and irretrievable data and a complex diversity of study design and indices used to measure the outcome parameters. Accordingly, only a descriptive analysis was performed, which is presented in Table 3.

**TABLE 3:** A descriptive summary of reported statistical significance in the original studies concerning NaOCl‐MW as compared to water or chlorhexidine‐MW

Design	Study #	Intervention	PI	GI	BI	PPD	Comparison	Positive/Negative Control
Non‐brushing protocol Experimental gingivitis model	I De Nardo et al. (2012) ^26^	0.05% NaOCl	+	+	+	NA	Water	Negative
Brushing protocol Gingivitis	II Espindola et al. (2017) ^28^	0.1% NaOCl	0	NA	0	0	Water	
Brushing protocol Periodontitis	V Galvan et al. (2014) ^39^ Gonzales et al. (2015) ^40^	0.25% NaOCl	+	NA	+	0	Water	
In summary			2/3+	1/1+	2/3+	0/2+		
Brushing protocol Gingivitis	III Shanker et al. (2018) ^27^	0.25% NaOCl	+	0	NA	NA	0.2% CHX	Positive
Brushing protocol Gingivitis	IV Mishra et al. (2019) ^25^	0.5% NaOCl	0	0	0	NA	0.2% CHX	
Brushing protocol Periodontitis	VI Singh et al. (2020) ^41^	0.05% NaOCl	0	NA	0	+	0.12% CHX	
In summary			1/3+	0/2+	0/2+	1/1+		

Abbreviations: +, significant difference in favour of the NaOCl group; 0, no significant difference; BI, bleeding index; CHX, chlorhexidine; GI, gingival Index; NA, not applicable; NaOCl, sodium hypochlorite; PI, plaque index; PPD, probing pocket depth.

#### 3.8.1 Comparisons with a negative control

Two of the three comparisons that used water as a negative control (I,^26^ V^39^, ^40^) showed statistically significant results for PI and BI scores in favour of NaOCl‐MW. Only one comparison (I^26^) assessed the GI and found this parameter to be statistically significant in favour of NaOCl‐MW. In two comparisons that measured PPD (II^28^ and V^39^, ^40^), no statistically significant difference was observed.

#### 3.8.2 Comparisons with a positive control

In studies III,^27^ IV^25^ and VI,^41^ which used CHX‐MW as a positive control, no statistically significant difference was found for the parameters GI and BI. One of the three studies (III^27^) showed a statistically significant PI score in favour of NaOCl‐MW, indicating that NaOCl‐MW is more effective than CHX‐MW. One study (VI^41^) measured PPD and found it to be statistically significant in favour of NaOCl‐MW.

### 3.9 Evidence profile

Table 4 presents a summary of the various aspects that were used to rate the quality of the evidence and to assess the strength and direction of the recommendations. These are presented separately for the negative and positive control studies. The risk of bias varied across the studies from low to high, and there was potential reporting bias. The data from the negative control group comparisons were ‘rather inconsistent’, ‘rather generalizable’ and ‘rather imprecise’. The effect of NaOCl‐MW compared with water was very small in favour of NaOCl‐MW. Altogether, the strength of the recommendation was estimated to be very weak for a very small effect favouring NaOCl‐MW over a negative control MW. When the participants used CHX‐MW as a positive control, the data were considered to be ‘rather consistent’, ‘rather generalizable’ and ‘rather imprecise’. There was no difference between CHX‐MW and NaOCl‐MW. Given the strength of the recommendation, there is very weak certainty that NaOCl‐MW is as effective as CHX‐MW and can be used as an alternative.

**TABLE 4:** Summary of findings table based on the quality and body of evidence on the estimated evidence profile and appraisal of the strength of the recommendation regarding the efficacy of NaOCl‐MW as compared to water or CHX‐MW (gold standard)

Determinants of the Quality	Water—Negative control	Chlorhexidine—Positive control
Study design	RCT	RCT/CCT
# studies, n=7 # comparisons n=6	# 4 # 3	# 3 # 3
Risk of bias (methodological limitations)	Low to high	Moderate to high
Consistency	Rather inconsistent	Rather consistent
Directness	Rather generalizable	Rather generalizable
Precision	Rather imprecise	Rather imprecise
Reporting bias	Possible	Possible
Magnitude of the effect	Very Small	No difference
Strength of the recommendation based on the quality and body of evidence	Very weak	Very weak
Direction of recommendation whether NaOCl‐MW can be used for the management of periodontal diseases	Very weak certainty for very small effect favouring NaOCl‐MW over a negative control MW	Very weak certainty for no difference between NaOCl‐MW as an alternative for CHX MW

Abbreviations: CCT, controlled clinical trial; CHX, chlorhexidine; MW, mouthwash; NaOCl, sodium hypochlorite; RCT, randomized controlled trial.

## 4 DISCUSSION

### 4.1 Summary of key findings

The purpose of this systematic review was to investigate the effect of rinsing with NaOCl‐MW in comparison with a positive or negative control mouthwash on plaque and clinical parameters of periodontal disease. This is the first review to our knowledge to systematically aggregate the evidence on NaOCl‐MW. Among the seven studies presenting six comparisons, considerable heterogeneity was observed regarding methodological and clinical aspects. The descriptive analysis based on the data from the included studies suggests that NaOCl‐MW reduces plaque scores and has a positive effect on the parameters of periodontal inflammation. Three comparisons (I,^26^ II^28^ and V^39^, ^40^) concerning NaOCl‐MW and a negative control group (Water) showed an inconsistent pattern, with two short‐term comparisons (I^26^ and V^39^, ^40^) showing significantly lower PI, GI, and BI scores and one long‐term comparison (II^28^) showing no significant difference. None of the comparisons showed an effect on PPD. Three comparisons (III,^27^ IV^25^ and VI^41^) that assessed NaOCl‐MW and a positive control group (CHX‐MW) showed no difference in BI and GI scores, indicating that NaOCl‐MW is as effective as CHX‐MW. One comparison (III^27^) showed NaOCl‐MW to be significantly more effective in reducing plaque scores, and another (VI^41^) showed a positive effect on PPD in patients with periodontitis.

### 4.2 Analysis

Owing to the heterogeneity of the indices used in the included studies and different study designs, it was impossible to combine the outcomes for a meta‐analysis. Instead, vote counting was used to synthesize the results of the selected studies. The Cochrane Handbook advises to limit vote counting to answer a simple question: *Is there any evidence of an effect?* ^29^ The study results were differentiated as non‐significant, significantly negative and significantly positive. With this classification, it was possible to combine the statistical analyses of the individual studies into an overall summary.^49^ The vote counting method, however, considers each study and each vote as equal and neither presents an estimate of the effect size of an intervention nor evaluates the precision.^50^ Hedges and Olkin affirm that vote counting is an appropriate method when only studies that show positive significant effect are considered.^51^ The data extracted for the present review were assessed accordingly. Positive outcomes of NaOCl‐MW on plaque scores and other parameters related to periodontal health were regarded in consideration of the estimate of the overall effect.

### 4.3 Outcome

The fact that one of the three comparisons with a negative control (II^28^) included in this systematic review showed no additional benefits of NaOCl‐MW on plaque and periodontal parameters can presumably be explained by differences in NaOCl concentrations, study design, periodontal condition of the participants selected for the study, as well as usage, frequency and duration of rinsing with the anti‐microbial mouthwash. Among the brushing studies that included patients with gingivitis, Espindola et al. (II^28^) used the lowest concentration 0.1%, as recommended by the American Dental Association (ADA). The other two studies, III^27^ and IV^25^ used a 2.5 and 5 times higher concentration, respectively, and in study IV,^25^ the patients used the mouthrinse for a duration twice as long. Furthermore, the patients in these studies (III^27^ and IV^25^) were instructed not to drink or eat for 30 min after rinsing, which means that the anti‐microbial agent could be present in the mouth for a longer period. Considering the outcome, it is likely that the ADA‐recommended concentration of 0.1% NaOCl does not provide the best anti‐microbial effect against the microflora related to periodontal disease, which may explain the findings reported by Espindola et al. (II^28^). However, comparison V^39^, ^40^ used a 2.5 times higher NaOCl concentration, but participants used the mouthrinse only twice a week. This essentially makes the exposure to the anti‐microbial agent comparable with the study by Espindola et al. (II^28^). However, the comparison also differed with respect to the periodontal condition of the participants and study design, which explains the positive effect. De Nardo et al. (I^26^) and Singh et al. (VI^41^) used the lowest concentration (0.05%) among all selected studies and still showed significant results favouring NaOCl. However, De Nardo et al. (I^26^) used an experimental gingivitis model,^52^ in which NaOCl‐MW was used as a substitute for regular oral hygiene measures. The results indicate that NaOCl prevents ‘de novo’ plaque formation. Furthermore, the study by Singh et al. (VI^41^) differed with respect to the periodontal condition as it included patients with periodontitis.

Two studies, including comparisons with a negative control group (II^28^ and V^39^, ^40^), measured the effect on PPD. No significant effect was observed. Comparison V^39^, ^40^ included patients with periodontitis, but non‐surgical periodontal therapy was only provided at the end of the study. Therefore, the absence of an effect was possibly due to the absence of mechanical instrumentation and also the limited number of study participants. Among the studies with a positive control group, only the study by Singh et al. (VI^41^) measured PPD and found it to be significant in favour of NaOCl‐MW. In this study, however, the participants received non‐surgical periodontal therapy in the beginning.

### 4.4 Side effects

The studies included in this review—except Singh et al. (VI^41^)—evaluated the potential side effects of NaOCl‐MW on hard and soft tissues. Three comparisons (I,^26^ II^28^ and V^39^, ^40^) used a special questionnaire; authors did not provide information on standardization of these questionnaires. The most frequently reported side effect in all studies was the unpleasant taste of bleach of the NaOCl‐MW immediately after rinsing. In studies I^26^ and V,^39^, ^40^ all participants reported the unpleasant taste, whereas in study II,^28^ 35% of the participants reported it. Furthermore, Espindola et al. (II^28^) reported altered taste (25%), and De Nardo et al. (I^26^) reported extrinsic brown tooth stains (100%), redness of the tongue (35%) and a burning sensation (45%). One side effect that was not addressed in any of the included studies was the bleaching effect of the rinsing solution if spilled, for instance, on clothing.

### 4.5 Hypochlorous acid

In water, NaOCl settles at an equilibrium with the strong active anti‐microbial agent, HOCl. HOCl is a weak acid and further dissociates into H^+^ and a less active anti‐microbial agent OCl^−^. The pH of the solution determines the concentration of HOCl. Thus, a high pH value ensures a high concentration OCl^−^ and a low concentration HOCl.^16^, ^17^ Household bleach is a basic solution (pH 11–12) causing the concentration of HOCl to be low.^53^ Furthermore, diluting a basic household bleach with an equal volume of water results in a different pH, leading to the changes in the anti‐microbial properties of diluted bleach.^16^ A more stable solution mainly containing hypochlorous acid (HOCl) can be made by electrolyzing water with salt.^16^ Two short‐term studies have been conducted with such a mouthwash solution.^54^, ^55^ Lafauri et al. (2018)^54^ compared HOCl‐MW (0.025% and 0.05%) with CHX‐MW (0.12% and 0.2%). No significant difference was found in PI scores after 7 h. Kim & Nam (2018)^55^ measured PI scores immediately after mouth rinsing to compare HOCl‐MW (0.001–0.003%) with CHX‐MW (0.005%). Significantly lower PI scores were found in the HOCl group than in the CHX group, indicating that HOCl‐MW was potentially superior. As these short‐term studies assessed the effect on initial plaque formation, longitudinal trials are required to explore the subsequent effects of HOCl‐MW on plaque and parameters of gingival inflammation.

### 4.6 Safety of NaOCl

The thought of using household bleach as a mouthwash may be an issue of concern for some patients who consider it harmful. The ADA approval of an over‐the‐counter NaOCl‐MW would likely reassure patients regarding its safety and efficacy. However, it is questionable whether such a product is attractive from a commercial perspective as people could use household bleach in a diluted form. Household bleach contains (according to manufacturers) water, NaOCl, sodium chloride (stabilizes formula), sodium carbonate (maintains alkalinity), sodium chlorate (is a process by‐product), sodium hydroxide (pH‐adjuster) and sodium polyacrylate (assists in cleaning)..^56^, ^57^ Two trials included in this review used Clorox in a diluted form and did not find any harmful side effects.^27^, ^39^, ^40^ Moreover, the ADA has proposed 0.1% NaOCl as a topical antiseptic for irrigation of wounds and as a mouthwash.^22^ Furthermore, Kalkwarf et al. (1982)^21^ studied the histological effect of the subgingival application of 5.3% NaOCl solution, and Perova et al. (1990)^58^ used it during periodontal surgery to disinfect the wound area with exposed alveolar bone. In both studies, no adverse effects were observed at the histological level. Thus, concentrations as low as 0.01–0.5%, similar to those used in the included studies, are presumably safe. Nevertheless, manufacturers should consider producing a NaOCl‐MW with a better taste that masks the bleach taste as it would encourage patient compliance. ^25^, ^26^

### 4.7 Limitations

This review has certain limitations. Specifically, the observed heterogeneity with respect to the study design and risk of bias makes it challenging to make a recommendation that is more than an expert opinion. Moreover, the English language criterion may have introduced a language bias. However, over the years, the extent and effects of such a possible bias have diminished because of the shift towards publication in the English language.^59^

### 4.8 Recommendation for further research

A meta‐analysis could not be performed on the studies that were included in this systematic review. To assist dental care professionals in providing evidence‐based recommendations for an NaOCl‐containing anti‐microbial mouthwash, there is a need of studies more homogeneous in terms of study design, NaOCl concentration, periodontal conditions of the patients, rinsing procedure, and indices used to measure plaque and periodontal parameters. In the future, this would allow for a meta‐analysis that takes the data one step further than the present descriptive analysis. Additionally, it appears of interest to evaluate a dose‐response effect of different NaOCl concentrations in a single RCT in which the side effects, and thus patient comfort, are also assessed more precisely. Two included clinical trials used household bleach as the source of NaOCl. As using household bleach could be an issue of concern, it would be interesting to investigate the effects of the other main ingredients of household bleach in low concentrations on oral soft and hard tissues. This information would assure people about the safety of using household bleach; it would be valuable especially for low‐income individuals as they are mostly at elevated risk for periodontal diseases ^60^, ^61^ because they lack the education on personal oral hygiene and are unable to afford oral care products of recognized brands.^62^ Therefore, there is a need to implement efficacious and low‐cost dental care products. NaOCl, which is widely available as household bleach, could be a low‐cost alternative.^24^, ^60^

## 5 CONCLUSION

Studies with a negative control group provided very weak quality evidence for a very small beneficial effect of NaOCl‐MW on PI, GI and BI scores. Studies with a positive control group provided very weak quality evidence that NaOCl‐MW had a similar effect as CHX‐MW on PI, GI and BI scores. The outcome for PPD was inconclusive.

## 6 CLINICAL RELEVANCE

### 6.1 Scientific rationale for the study

Most individuals cannot achieve efficient mechanical plaque control. Thus, adjunctive use of anti‐microbial agents may be required. NaOCl has been proposed as an inexpensive mouthwash for long‐term use.

### 6.2 Principal findings

Compared with a negative control, NaOCl‐MW showed a significant effect on PI, GI and BI scores. Summary data of the comparisons with CHX‐MW as a positive control suggested no significant difference.

### 6.3 Practical implications

There is very weak quality evidence that household bleach in a diluted form can be prescribed as adjunct to mechanical cleaning to prevent or treat plaque and gingivitis.

## CONFLICT OF INTEREST

This study was in part prepared as obligation of first author to fulfil the requirements of the ACTA master programme of Dentistry. Hussain declares no conflicts of interest. Van der Weijden, Slot and their research team at ACTA have previously received either external advisor fees, lecturer fees or research grants from dental care product manufacturers. Those manufacturers included GABA/Colgate, Dentaid, Lactona, Oral‐B/Procter & Gamble, Sara Lee, Sunstar Philips Unilever, GSK, Listerine and Waterpik. This research received no specific grant from any funding agency in the public, commercial, or not‐for‐profit sectors. For this study, no funding was accepted, except for support from the listed institution. The work for this paper was performed by the regular academic appointments of Slot and Van der Weijden at the Academic Centre for Dentistry Amsterdam (ACTA).

## AUTHOR CONTRIBUTIONS

AMH contributed to design, search and selection, analysis and interpretation, and drafted the manuscript. DES contributed to conception and design, search and selection, analysis and interpretation, and critically revised the manuscript. GAW contributed to conception and design, analysis and interpretation, and critically revised the manuscript. All authors gave final approval and agreed to be accountable for all aspects of work ensuring integrity and accuracy.

## ETHICAL APPROVAL

Ethical approval was not required, and the protocol was registered at ACTA ETC (protocol number 202093).

## Supporting information

Supplementary Material

## Data Availability

Data derived from public domain resources. The data that support the findings (the seven included studies) of this study are available from search databases PubMed/Medline, Cochrane‐CENTRAL or EMBASE. These data were derived from resources available in original papers that are published in the public domain.
