# Supplementary material for: Effect of a sodium hypochlorite mouthwash on plaque and clinical parameters of periodontal disease‐a systematic review
Source: Int J Dent Hyg. 2021 Jul 19;20(1):40–52. doi: 10.1111/idh.12510 (PMC9292655; doi:10.1111/idh.12510)
Supplement: Supplementary file 1 — Supplementary Material [file IDH-20-40-s001.pdf]

Effect of a sodium hypochlorite mouthwash on plaque and clinical parameters of periodontal disease

-A Systematic Review-

A.M. Hussain

G.A. van der Weijden

D.E. Slot

Online appendices

Online Supporting information legends

Online Appendix S1.

Methodological quality and potential risk of bias scores of the individual included studies.

Online Appendix Mean (SD) scores for the different intervention groups

Online Appendix S2 Plaque

Online Appendix S3 Gingival index

Online Appendix S4 Bleeding index

Online Appendix S5 Probing pocket depth

ONLINE Appendix S1.

Methodological quality and potential risk of bias scores of the individual included studies

		De Nardo et al. (2012)[26]	Espindola et al. (2017)[28]	Galvan et al. (2014)[39] Gonzales et al. (2015)[40]	Shanker et al. (2018)[27]	Mishra et al. (2019)[25]	Singh et al. (2020)[41]
		**Non brushing**<br>**Experimental gingivitis model**	**Brushing**<br>**Gingivitis**	**Brushing**<br>**Periodontitis**	**Brushing**<br>**Gingivitis**	**Brushing**<br>**Gingivitis**	**Brushing**<br>**Periodontitis**
	Study design	RCT (parallel)	RCT (parallel)	RCT (parallel)	RCT (parallel)	CCT (parallel)	RCT (parallel)
**internal validity**	Random allocation *	+	+	+	+	-	+
	Allocation concealment	?	+	?	?	-	?
	Blinded to intervention *	-	+	-	?	?	?
	Blinded to operator *	+	+	+	+	?	?
	Blinding during statistical analysis	?	+	?	?	?	?
	Balanced experimental groups *	?	+	+	+	?	?
	Reported loss to follow up*	+	+	+	+	-	-
	# (%) of dropouts	4 (9%)	4 (8,9%)	18(60%)	20 (20%)	?	?
	Treatment identical, except for intervention *	+	+	+	+	+	+
	Representative population group	-	+/-	+/-	+/-	+/-	?
	Eligibility criteria defined *	+	+	+	+	+	-

	Sample size calculation and power	-	+	?	-	+	?
	Point estimates presented for the primary outcome	+	+/-	+	+	+	+
	Measures of variability presented for the primary outcome	+/-	+/-	+	-	+	+
	Include a per protocol analysis	+	+	+	+	-	-
	Include an intention- to-treat analysis	-	-	-	-	-	-
Clinical aspects	Validated measurement	+	?	?	+	+	+
	Calibration examiner	+	+	+	?	?	?
	Trained operator	+	+	+	+	?	?
	Reproducibility data shown	+	-	+	-	-	-
**Authors estimated risk of bias**		**High**	**Low**	**Moderate**	**Moderate**	**High**	**High**

Each aspect of the score list was given a rating of '+' for an informative description of the item concerned and a study design meeting the quality standard, '-' for an informative description without a study design that met the quality standard, and '?' for lacking or insufficient information. When random allocation, defined eligibility criteria, blinding of operator and patients, balanced experimental groups, identical treatment between groups (except for intervention), and report of follow-up were present, the study was classified as having a low risk of bias. When one of these seven criteria were missing, the study was considered to have a moderate potential risk of bias. When two or more of these criteria were missing, the study was considered to have a high potential risk of bias, as proposed by Van der Weijden et al. (2009)[34]

Abbreviations:
? = not specified/unclear; + = yes; - = no; * = reporting criteria for estimation the potential risk of bias; RCT=randomized controlled trial, CCT=clinical controlled trail

ONLINE Appendix S2. Plaque

Mean (SD) scores for the different intervention groups with various indices and their modifications.

Design	#	Index	Intervention groups		Mean (SD)		
			Product	%	Baseline	End	Difference
Non brushing<br>*Experimental gingivitis model*	**I**<br>**De Nardo et al. (2012)**[26]	Turesky et al. (1970)[44] modification of the Quigley & Hein (1962)[43]	NaOCl<br>Water	**0.05%**	0 (0)<br>0 (0)	1.98 (0.51)<br>3.82 (0.61)	-1.98 (0.51) □<br>-3.82 (0.61) □
Brushing<br>Gingivitis	**II**<br>**Espindola et al. (2017)**[28]	Presence or absence at six sites<br>% sites with plaque	NaOCl<br>Water	**0.1%**	34.85% (17.2%)<br>31.85% (8.9%)	18.20% (7.52%) □<br>17.75% (5.31%) □	16.65% (16.98%) □<br>14.10% (9.88%) □
	**III**<br>**Shanker et al. (2018)**[27]	Turesky et al. (1970)[44] modification of the Quigley & Hein (1962)[43]	NaOCl<br>CHX	**0.25%**<br>**0.2%**	0 (0) □<br>0 (0) □	2.32 (??)<br>3.62 (??)	-2.32 (??) □<br>-3.62 (??) □
	**IV**<br>**Mishra et al. (2019)**[25]	Löe & Silness (1963)[45]	NaOCl<br>CHX	**0.5%**<br>**0.2%**	1.77 (0.21)<br>1.78 (??)	0.51 (0.11)<br>0.50 (??)	1.26 (??)<br>1.28 (??)

**Brushing** **Periodontitis**	**V** **Galvan et al. (2014)**[39] **Gonzales et al. (2015)**[40]	Presence or absence at two sites (facial and lingual surface) % plaque free sites	NaOCl Water	**0.25%**	Facial 38% (29%) Lingual 18% (23%) Facial 34% (30%) Lingual 30% (33%)	Facial 74% (17%) Lingual 53% (33%) Facial 44% (30%) Lingual 39% (23%)	-36% (??) -35% (??) -10% (??) -9% (??)
	**VI** **Singh et al. (2020)**[41]	Löe & Silness (1963)[45]	NaOCl CHX	**0.05%** **0.12%**	2.47 (0.51) 2.57 (0.50)	0.87 (0.43) 0.83 (0.46)	1.60 (??) 1.73 (??)

Abbreviations: NaOCl= Sodium hypochlorite; CHX= Chlorhexidine; ? = Unknown/not provided; □ = Data requested from original authors, SD= standard deviation

ONLINE Appendix S3. Gingival index

Mean (SD) scores for the different intervention groups with various indices and their modifications.

Design	#	Index	Intervention groups		Mean (SD)		
			Product	%	Baseline	End	Difference
Non brushing<br>*Experimental gingivitis model*	**I**<br>**De Nardo et al. (2012)**[26]	Löe & Silness (1963)[45]	NaOCl<br>Water	**0.05%**	0.12 (0.05)<br>0.08 (0.04)	1.00 (0.2)<br>2.10 (0.2)	-0.88 (0.22) □<br>-2.02 (0.22) □
Brushing<br>Gingivitis	**III**<br>**Shanker et al. (2018)**[27]	Modified Gingival Index<br>Lobene et al.(1986)[46]	NaOCl<br>CHX	**0.25%**<br>**0.2%**	0 (0) □<br>0 (0) □	1.20 (??)<br>1.22 (??)	-1.20 (??) □<br>-1.22 (??) □
	**IV**<br>**Mishra et al. (2019)**[25]	Löe & Silness (1963)[45]	NaOCl<br>CHX	**0.5%**<br>**0.2%**	1.81 (0.26)<br>?? (??)	0.62 (0.13)<br>0.60 (??)	1.19 (??)<br>?? (??)

Abbreviations: NaOCl= Sodium hypochlorite; CHX= Chlorhexidine; ? = Unknown/not provided; □ = Data requested from original authors, SD= standard deviation

ONLINE Appendix S4. Bleeding index

Mean (SD) scores for the different intervention groups with various indices and their modifications.

Design		#	Index	Intervention groups		Mean (SD)		
				Product	%	Baseline	End	Difference
**Non brushing**	***Experimental gingivitis model***	**I**<br>**De Nardo et al. (2012)**[26]	Probing to the bodem of the pocket at four sites (mesiofacial, midfacial, distofacial, midlingual) % sites with bleeding	NaOCl<br>Water	**0.05%**	7.7% (??)<br>7.3% (??)	56.7% (??)<br>93.1% (??)	-49% (??) □<br>-85.8% (??) □
**Brushing**	**Gingivitis**	**II**<br>**Espindola et al. (2017)**[28]	Measured at six sites<br>% sites with bleeding	NaOCl<br>Water	**0.1%**	19.59% (6.9%)<br>22.0% (8.3%)	8.84% (3.95%) □<br>7.86% (2.41%) □	10.74% (6.03%) □<br>14.14% (7.48%) □
		**IV**<br>**Mishra et al. (2019)**[25]	Bleeding on marginal probing<br>Ainamo & Bay (1975)[47]	NaOCl<br>CHX	**0.5%**<br>**0.2%**	93.20 (6.84)<br>94.16 (??)	33.53 (6.56)<br>33.69 (??)	59.67 (??)<br>60.47 (??)
**Brushing**	**Periodontitis**	**V**<br>**Galvan et al. (2014)**[39]<br><br>**Gonzales et al. (2015)**[40]	Probing to the bodem of the pocket at six sites % bleeding free teeth	NaOCl<br>Water	**0.25%**	12% (18%)<br>31% (32%)	62% (29%)<br>40% (45%)	-50% (??)<br>-9% (??)

	**VI Singh et al. (2020)**[41]	Gingival sulcus bleeding Mühlemann, H.R. and Son, S. (1971)[48]	NaOCl<br>CHX	**0.05%**<br>**0.12%**	2.43 (0.50)<br>2.47 (0.51)	0.30 (0.47)<br>0.53 (0.57)	2.13 (??)<br>1.93 (??)

Abbreviations: NaOCl= Sodium hypochlorite; CHX= Chlorhexidine; ? = Unknown/not provided; □ = Data requested from original authors, SD= standard deviation

ONLINE Appendix S5. Probing pocket depth

Mean (SD) scores for the different intervention groups with various indices and their modifications.

Design	#	Index	Intervention groups		Mean (SD)		
			Product	%	Baseline	End	Difference
Brushing Gingivitis	**II Espindola et al. (2017)[28]**	Measured at six sites (mesiofacial, midfacial, distofacial, mesiolingual, midlingual, distolingual)	NaOCl<br>Water	**0.1%**	1.95 (0.13)<br>1.97 (0.12)	2.16 (0.16) □<br>2.18 (0.11) □	-0.20 (0.16) □<br>-0.21 (0.13) □
Brushing Periodontitis	**V Galvan et al. (2014)[39]**<br><br>**Gonzales et al. (2015)[40]**	Measured at six sites (mesiofacial, midfacial, distofacial, mesiolingual, midlingual, distolingual)	NaOCl<br>Water	**0.25%**	?? (??)<br>?? (??)	?? (??)<br>?? (??)	?? (??)<br>?? (??)
	**VI Singh et al. (2020)[41]**	Measured at six sites (mesiofacial, midfacial, distofacial, mesiolingual, midlingual, distolingual)	NaOCl<br>CHX	**0.05%**<br>**0.12%**	5.67 (0.84)<br>5.57 (0.90)	2.17 (0.38)<br>2.50 (0.68)	3.50 (??)<br>3.07 (??)

Abbreviations: NaOCl= Sodium hypochlorite; CHX= Chlorhexidine; ? = Unknown/not provided; □ = Data requested from original authors, SD= standard deviation
